# Ewing’s Sarcoma Mimicking an Avulsion Fracture

**DOI:** 10.5334/jbsr.2618

**Published:** 2021-11-22

**Authors:** Charlotte Vanhoenacker, Jan Myncke, Filip Vanhoenacker

**Affiliations:** 1 UZ Leuven, BE; 2 H.H. Hospital Lier, BE; 3 AZ Sint-Maarten and University (Hospital) Antwerp/Ghent, BE

**Keywords:** Ewing’s sarcoma, avulsion fracture of the lesser trochanter, MRI, CT, radiography

## Abstract

**Teaching Point:** In the absence of a clear history of trauma, avulsion of the lesser trochanter should raise a high index of suspicion of an underlying malignancy.

## Case Presentation

**Figure 1.**
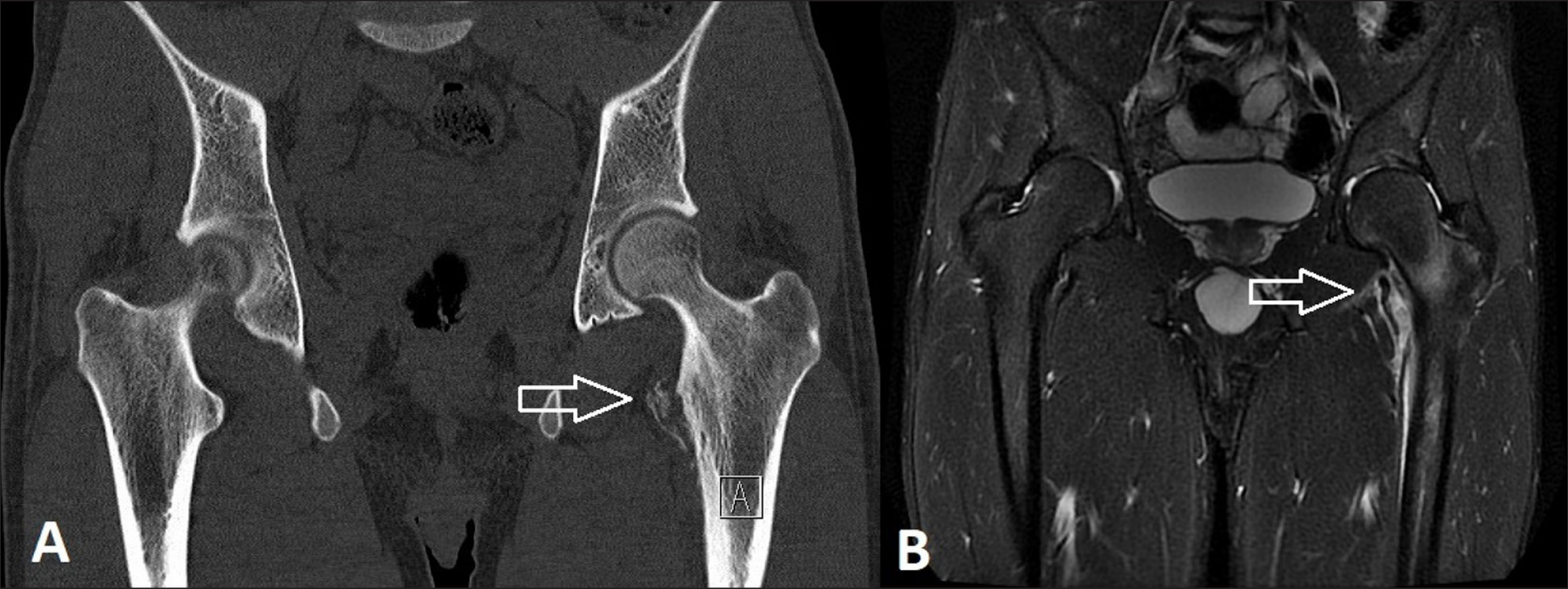


**Figure 2.**
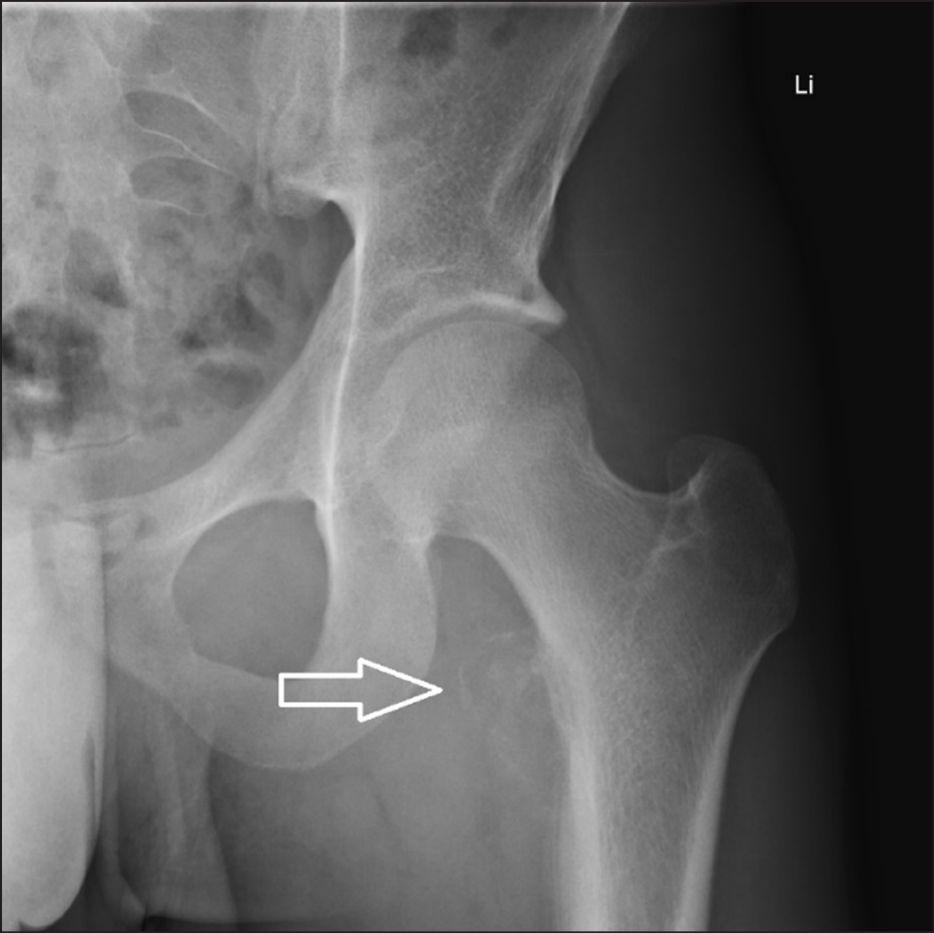


**Figure 3.**
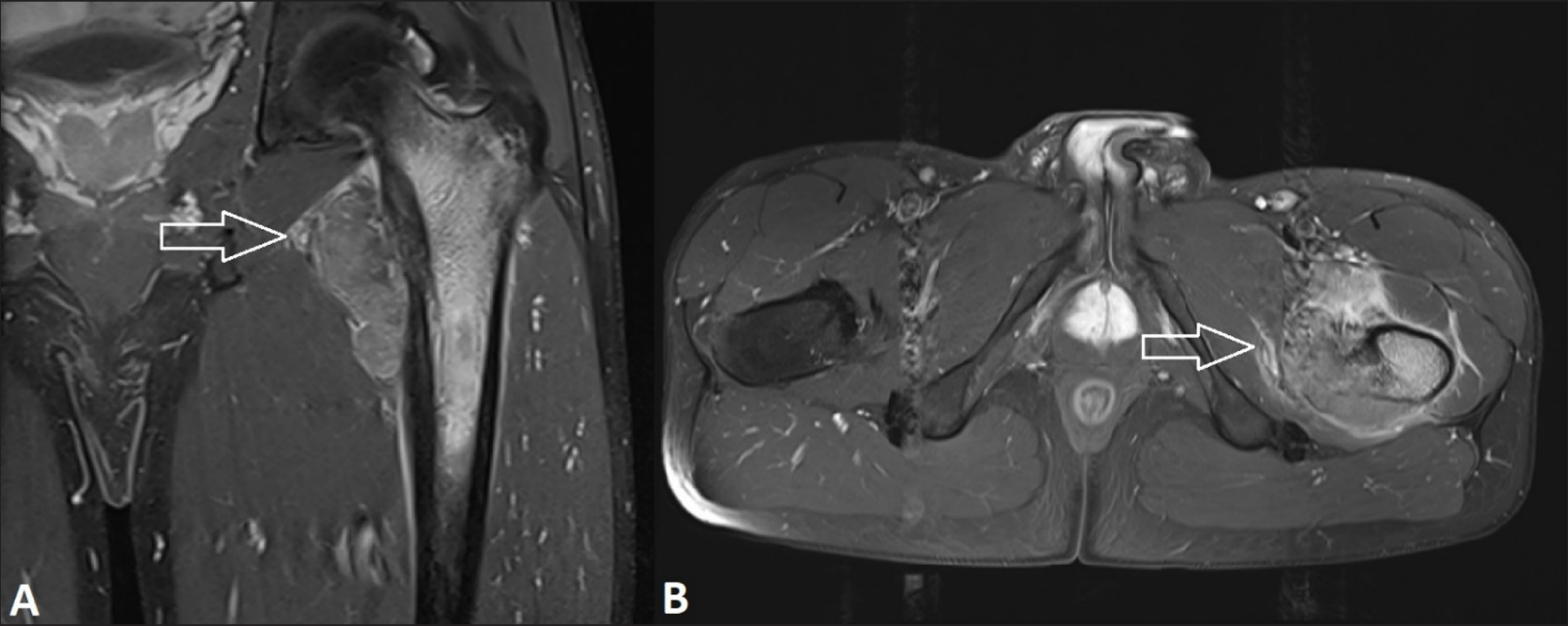


An 18-year-old cyclist was admitted to our hospital for spontaneous onset pain in the left groin for the past months. Computed tomography (CT) showed a fracture at the lesser trochanter of the left femur, which was confirmed on the subsequent magnetic resonance imaging (MRI) (***Figure 1***, arrows). The initial diagnosis of an avulsion fracture of the lesser trochanter was made, in accordance with the professional activities of the patient. Because of aggravating pain and the onset of nightly pain, a follow-up radiograph was performed a few weeks later, showing an expansile radiolucency of the lesser trochanter with an adjacent layered periosteal reaction at the medial cortex of the proximal femoral diaphysis (***Figure 2***, arrow). These findings were suggestive of an aggressive osseous lesion. Repeated MRI showed an osseous lesion causing destruction of the medial cortical bone and a large soft tissue component posteromedial the proximal femoral diaphysis. There was heterogeneous enhancement of the mass (***Figure 3***, arrows). Perilesional edema was present in the iliopsoas and vastus intermedius muscles. The combination of the age of the patient, the absence of a history of trauma, and the aggressive appearance of the osseous lesion with a large soft tissue component was suspicious for Ewing’s sarcoma. After histopathological confirmation, the lesion was treated with neoadjuvant chemotherapy, followed by resection of the proximal femur shaft and placement of a femoral prosthesis. Further follow-up was uneventful.

## Comment

Ewing’s sarcoma is a highly aggressive neoplasm and the second most common bone tumor in children and adolescents with a peak incidence between 10–20 years. The metaphysis of the long bones is most involved (80%), and the femur is the most affected (25%) [1].

Location in the lesser trochanter may rarely mimic an avulsion fracture. The imaging appearance of Ewing’s sarcoma is highly variable and often presents as a large permeative lesion, with lamellated periosteal reaction. Conventional radiography shows an aggressive osteolytic lesion. Computed tomography (CT) is more sensitive for the detection of bony destruction in more complex regions. Magnetic resonance imaging (MRI) is the modality of choice for evaluating local tumor extension and staging. Typically, there is bone marrow replacement with heterogeneous enhancement and a large soft tissue mass. Systemic chemotherapy is the keystone in treatment; additional surgery or radiotherapy can be performed depending on the location and size of the lesion.

Underlying malignancy should be excluded in case of avulsion fracture of the lesser trochanter, particularly in the absence of trauma.
